# A sellar presentation of a WNT-activated embryonal tumor: further evidence of an ectopic medulloblastoma

**DOI:** 10.1186/s40478-023-01556-3

**Published:** 2023-04-03

**Authors:** Arnault Tauziède-Espariat, Marie Simbozel, Anthony P. Y. Liu, Giles W. Robinson, Julien Masliah-Planchon, Philipp Sievers, Alexandre Vasiljevic, Mathilde Duchesne, Stéphanie Puget, Volodia Dangouloff-Ros, Nathalie Boddaert, Alice Métais, Lauren Hasty, Christelle Dufour, Pascale Varlet

**Affiliations:** 1 Department of Neuropathology, GHU Paris-Psychiatrie et Neurosciences, Sainte-Anne Hospital, 1, Rue Cabanis, 75014 Paris, France; 2 Inserm, UMR 1266, IMA-Brain, Institut de Psychiatrie et Neurosciences de Paris, Paris, France; 3 Department of Child and Adolescents Oncology, Gustave Roussy, Université Paris-Saclay, 94805 Villejuif, France; 4 Department of Paediatrics and Adolescent Medicine, Li Ka Shing Faculty of Medicine, The University of Hong Kong, Hong Kong SAR, China; 5 Department of Oncology, St. Jude Children’s Research Hospital, Memphis, TN USA; 6 Laboratory of Somatic Genetics, Institut Curie Hospital, 75248 Paris Cedex 5, France; 7 Department of Neuropathology, Institute of Pathology, University Hospital Heidelberg, Heidelberg, Germany; 8 Clinical Cooperation Unit Neuropathology, German Consortium for Translational Cancer Research (DKTK), German Cancer Research Center (DKFZ), Heidelberg, Germany; 9 Department of Pathology and Neuropathology, GHE, Hospices Civils de Lyon, Lyon, France; 10 Department of Pathology, Dupuytren University Hospital, Limoges, France; 11 Department of Paediatric Neurosurgery, Necker Hospital, APHP, Université Paris Descartes, Sorbonne Paris Cité, 75015 Paris, France; 12 Pediatric Radiology Department, Hôpital Necker Enfants Malades, AP-HP, Paris, France; 13 UMR 1163, Institut Imagine and INSERM U1299, Université Paris Cité, Paris, France

Over the past several years, medulloblastomas have been subdivided into four different subgroups based on genetic and epigenetic data (WNT-activated, SHH-activated, and groups 3 and 4) [7]. It has been evidenced that these subgroups are correlated to their precise anatomic location in the posterior fossa (cerebellar hemispheres *vs.* dorsal brainstem) and their cell of origin [5]. However, a recent study reported 7 cases of WNT-activated embryonal tumors epicentered in the pineal region that clustered within the DNA-methylation class of medulloblastomas, WNT-activated (MB-WNT) [6]. These results suggest the possibility of a potential novel pineoblastoma subgroup or an ectopic location of medulloblastoma.

Herein, we report the case of a previously healthy 5-years-old girl who presented with visual loss. Magnetic resonance imaging (MRI) revealed a 6 cm solid enhancing mass located in the sellar and supra-sellar regions (Fig. 1A–C). There was no other lesion in the CNS (including the cerebellum, the pineal gland, and leptomeninges; and no evidence of a developmental abnormality elsewhere in the brain). Cerebrospinal fluid cytology did not evidence tumor cells. The first biopsy of the lesion showed an atrophic cerebellar parenchyma with Purkinje neurons and granular cells (Fig. 1D, E). A few weeks later, intracranial hypertension symptoms appeared and a repeated MRI revealed tumor progression. A second biopsy was performed. This one showed an embryonal tumor composed of sheets of cells having numerous mitoses and apoptotic figures (Fig. 1F). No Homer-Wright or Flexner–Wintersteiner rosettes were present. Immunohistochemical analyses exhibited diffuse synaptophysin staining. INI1 and BRG1 stainings were maintained, and there was no immunoexpression of LIN28A. The tumor was initially classified as a primitive neuroectodermal tumor (PNET), because the diagnosis was made before 2016. The patient received different lines of chemotherapy (VP16-carboplatin and cyclophosphamide) and focal radiation therapy. The tumor residue was stable during follow-up, but the patient died due to infectious complications (pneumonia) 8 years after the initial diagnosis. The pituitary function was intact. A revised update of the initial diagnosis was recently performed. The tumor was CRX negative by immunohistochemistry (Fig. 1G). DNA-methylation profiling classified the tumor as MB-WNT (v12.5, with a calibrated score of 0.99). t-Distributed Stochastic Neighbor Embedding (t-SNE) analysis was undertaken for comparison to the genome-wide DNA methylation profiles of the CNS reference cohort (which included in particular, the different subgroups of medulloblastomas and pineal tumors) and the previously reported WNT-activated embryonal tumors of the pineal region [6]. Using unsupervised t-SNE, the tumor clustered with MB-WNT from the St Jude cohort of reference and their pineal WNT-activated counterparts (Fig. 1H) [3]. An orthogonal validation was performed using a genetic (detection of a c.104T > G/p.(Ile35Ser) mutation of the *CTNNB1* gene) and immunohistochemical analyses (presence of nuclear β-catenin accumulation, YAP1 immunopositivity without GAB1 expression in tumor cells) (Fig. 1I and Additional file 1: Fig. S1).

**Fig. 1:**
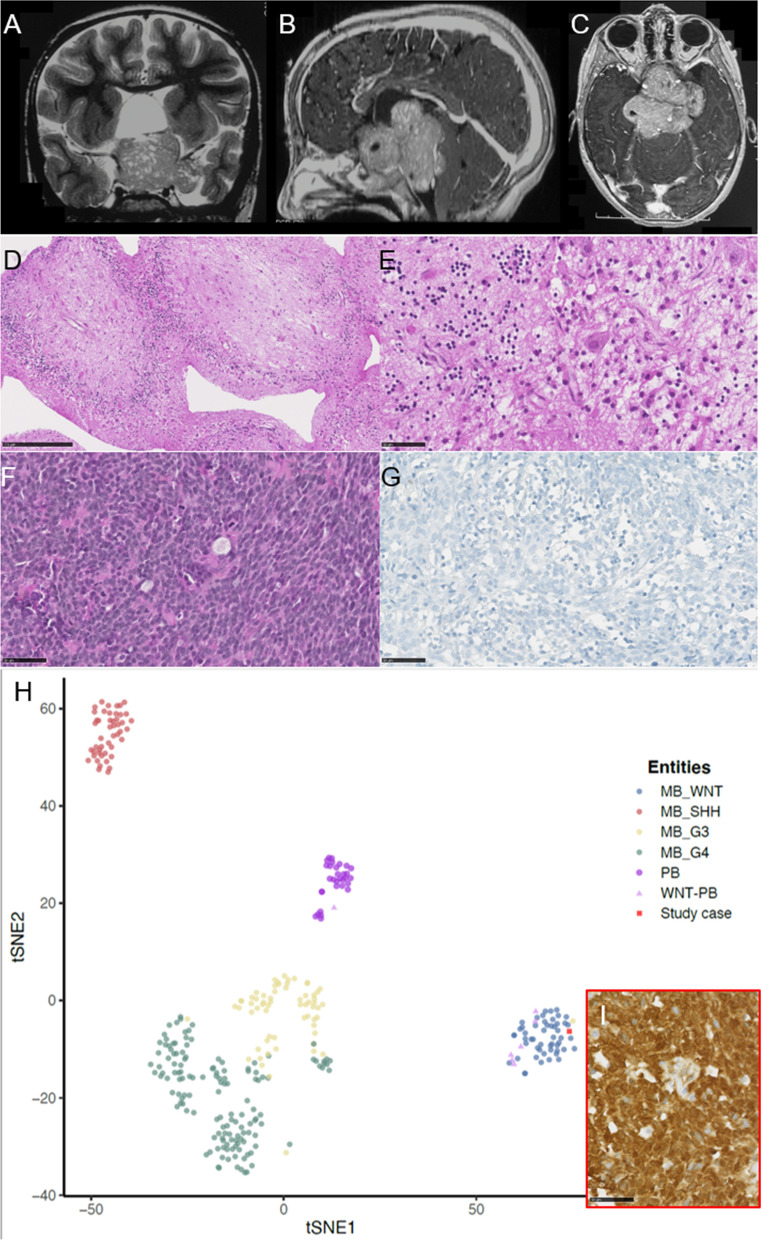
Radiological, and histopathological features and t-distributed stochastic neighbor embedding (t-SNE) analysis of DNA methylation profiles of the investigated tumor alongside selected reference samples. **A**, **B**, **C** Magnetic resonance imaging revealing a lobulated sellar and supra-sellar mass, with enhancing tissular content, microcysts and a pronounced mass effect on the brainstem. **D** First biopsy showing atrophic cerebellar parenchyma (HPS, ×200 magnification) with **E** Purkinje neurons and granular cells (HPS, ×400 magnification). **F** The second biopsy showed an embryonal tumor (HPS, ×400 magnification). **G** No expression of Crx (×400 magnification). **H** Reference DNA methylation classes: medulloblastomas, WNT-activated (MB_WNT); medulloblastomas, SHH-activated (MB_SHH); medulloblastomas, group 3 (MB_G3); medulloblastomas, group 4 (MB_G4); pineoblastomas (PB); previously reported embryonal tumors, WNT-activated of the pineal gland (WNT-PB); and the current case. **I** Nuclear accumulation of β-catenin (×400 magnification). Black scale bars represent 250 μm (**D)** and 50 μm (**E**,** F**,** G**, and** I**)

Here, we report the first sellar presentation of an MB-WNT. Based on our results, we can argue that this exceptional case represents an ectopic location of the classically posterior fossa tumor. Indeed, for this case, there was no tumor in the posterior fossa, and the first biopsy showed a non-tumoral cerebellar parenchyma which may constitute the medulloblastoma cellular origin. Ectopy of cerebellar tissue was previously reported in different regions of the brain [1, 3, 4, 8], including the sellar region [2], for which a potential differential diagnosis could be a teratoma with a somatic-type malignancy. However, no other mature or immature tissular component was evidenced for this case, which shared the same DNA-methylation profile as previously reported pineal forms of WNT-activated embryonal tumors and MB-WNT located in the posterior fossa. DNA methylation profiles are thought to represent a combination of both somatically acquired DNA methylation changes and a signature reflecting the cell of origin. Consequently, it is reasonable to assume that our case represents an ectopic presentation of MB-WNT or a tumor originating from a migratory dysfunction of cerebellum progenitors during development. This case was located in the sellar region and no pineal involvement was observed. Interestingly, the reported WNT-activated embryonal tumors have been primarily located in the pineal gland but two cases were thalamic or affected the third ventricle. These different locations argue against a novel molecular subgroup of pineoblastoma, which is further supported by the absence of CRX immunoreactivity. Moreover, the current WNT alteration, representing one of the rarest hotspot mutations described in *CTNNB1*-mutated tumors, was not reported in the pineal cases.

To conclude, this case constitutes a sellar example of ectopic MB-WNT based on morphological, genetic and epigenetic analyses. Despite the molecular similarities of this case to its counterparts found in the posterior fossa and pineal gland, analyses have not been in favor of a potential novel molecular pineoblastoma subtype.

## Supplementary Information

**Additional file1**. **Figure S1**: Immunohistochemical findings. (A) Diffuse immunopositivity for YAP1 (x400 magnification). (B) No immunoreactivity for GAB1 (x400 magnification). Black scale bars represent 50 μm.
