# Supplementary figures and images for: A sellar presentation of a WNT-activated embryonal tumor: further evidence of an ectopic medulloblastoma
Source: Acta Neuropathol Commun. 2023 Apr 3;11:58. doi: 10.1186/s40478-023-01556-3 (PMC10069078; doi:10.1186/s40478-023-01556-3)

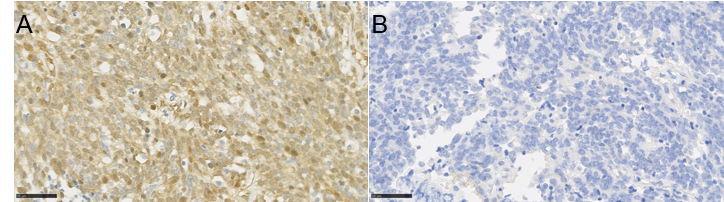

Supplement: Supplementary file 1 — Additional file1. Figure S1: Immunohistochemical findings. (A) Diffuse immunopositivity for YAP1 (x400 magnification). (B) No immunoreactivity for GAB1 (x400 magnification). Black scale bars represent 50 μm. [file 40478_2023_1556_MOESM1_ESM.tif]
